# Comment on “Considerations for the treatment of pancreatic cancer during the COVID-19 pandemic: the UK consensus position”

**DOI:** 10.1038/s41416-020-01132-9

**Published:** 2020-11-03

**Authors:** Ali Arshad, Ashley Dennison, Mark A. Taylor, Andrew Cook, John N. Primrose

**Affiliations:** 1 University Hospital Southampton, Southampton, UK; 2 HPB Surgery, University Hospitals of Leicester, Leicester, UK; 3 Pancreatic Society and the Great Britain and Ireland Hepatopancreatobiliary Association, London, UK; 4 HPB Surgery, Mater Hospital, Belfast, UK; 5 Great Britain and Ireland Hepatopancreatobiliary Association, London, UK; 6 Public Health Medicine, Wessex Institute, Faculty of Medicine, University of Southampton, Southampton, UK; 7 University of Southampton, Southampton, UK; 8 Association of Surgeons of Great Britain and Ireland, London, UK

**Keywords:** Health services, Health care

We refer to the publication by Jones et al.^1^ audaciously described as being “the UK consensus position” on the treatment of pancreatic cancer during the COVID-19 pandemic. A consensus statement certainly has no hard and fast definition. It can range from 2 people publishing the result of a casual discussion to an elaborate process involving DELPHI and AGREE methodology. Consensus statements using the latter approach do exist in the management of pancreatic cancer^2^ and major journals tend to publish these outputs, but not the former. As the paper states that it is the UK consensus it would be expected to have widespread engagement with the community treating pancreas cancer. Examining its provenance however, it remains unclear how the 18 members were selected. Ten of the 18 members are in fact clinical (radiation) oncologists, and radiation has a small role in the management of the disease compared to surgery (3 members) and chemotherapy (5 members). There is no radiologist or gastroenterologist, no palliative care physician, no pathologist, no specialist nurse or any other non-medical health care professional involved in the care of patients and, most important, no patient/consumer representation. Pancreas Cancer UK (a charity) were apparently asked to comment on the manuscript. It is, therefore, not obvious that these 18 members represent the relevant community. Turning to the review itself it is unclear what methodology was used to evaluate the evidence. At some points, but not generally, an evidence level is given (e.g. 2a, etc.). This resembles the system used in SIGN^3^ but there is no such reference in the paper. There is, therefore, no reason to be certain that a different 18 clinicians may not reach different conclusions faced with the current evidence. There is excessive detail on the radiation oncology management in the paper bearing in mind the very limited role of radiotherapy in the condition. One is left to consider whether the predominance of clinical (radiation) oncologists in the authorship has led to this singular feature. As regards the rest of the recommendations, they are now of little relevance even if they ever had merit. With universal testing of patients preoperatively combined with self- isolation, COVID free sites/pathways, and staff testing, there is no reason why the management of pancreas cancer should be different from prior to the pandemic. Further with better engagement the authors may have realised that many sites had very little disruption to the management of these cases even at the height of the pandemic. In summary authors aim who write “consensus statements” should first ensure that appropriate and well-described methodologies are followed and have enough modesty to be aware that they may not have the only views worthy of consideration. Similarly, major journals need to ensure that there should be a continuing focus on quality and robust peer review irrespective of the temptations afforded by the current pandemic.

## Data Availability

Not applicable.
